# Transgenic *Eimeria tenella* as a vaccine vehicle: expressing TgSAG1 elicits protective immunity against *Toxoplasma gondii* infections in chickens and mice

**DOI:** 10.1038/srep29379

**Published:** 2016-07-08

**Authors:** Xinming Tang, Guangwen Yin, Mei Qin, Geru Tao, Jingxia Suo, Xianyong Liu, Xun Suo

**Affiliations:** 1 State Key Laboratory of Agrobiotechnology & Key Laboratory of Zoonosis of Ministry of Agriculture & National Animal Protozoa Laboratory, College of Veterinary Medicine, China Agricultural University, Beijing, 100193, China; 2 Engineering Laboratory of Animal Pharmaceuticals, College of Animal Science, Fujian Agriculture and Forestry University, Fuzhou, 350002, Fujian Province, China

## Abstract

The surface antigen 1 of *Toxoplasma gondii* (TgSAG1) is a major immunodominant antigen and is widely considered an ideal candidate for the development of an effective recombinant vaccine against toxoplasmosis. *Eimeria tenella*, an affinis apicomplexan parasite with *T. gondii*, is a potential vaccine vector carrying exogenous antigens that stimulates specific immune responses. Here, we engineered TgSAG1 into *E. tenella* and obtained a stably transfected *E. tenella* line (Et-TgSAG1). We found TgSAG1 localized on the cell surface of Et-TgSAG1, which is similar to its native distribution in *T. gondii* tachyzoites. We immunized the chickens with Et-TgSAG1 orally and detected TgSAG1-specific immune responses, which partly reduced *T. gondii* infection. In the mouse model, we immunized the mice with Et-TgSAG1 sporozoites intraperitoneally and challenged them with *T. gondii* tachyzoites RH strain. We found that the mice immunized with Et-TgSAG1 showed a TgSAG1 specific Th 1-dominant immune response and a prolonged survival time compared with wild-type *E. tenella* and non-immunized mice. Collectively, our results demonstrated that Et-TgSAG1, utilized as a recombinant vaccine against toxoplasmosis, could be applied in both chickens and mice. Our findings also provide a promising persuasion for the development of transgenic *Eimeria* as vaccine vectors for use in birds and mammals.

*Toxoplasma gondii* is a ubiquitous pathogen with a worldwide distribution, and has caused infection in approximately one-third of the world’s human population^1^,^2^,^3^. *T. gondii* infection is generally asymptomatic in most immunocompetent individuals, but it may cause abortion, neonatal death, severe sequelae in neonates, and lethal encephalitis in immunocompromised patients, such as AIDS patients^3^,^4^,^5^. Infections are mainly acquired by the ingestion of food or water that is contaminated with oocysts shed by cats or by eating undercooked or raw meat (e.g., beef, pork, lamb and chicken) containing tissue cysts^3^,^6^,^7^.

Very high prevalence rates of *T. gondii* have been found in chickens raised in backyards (up to 100%) and free-range organic (30–50%) establishments, although toxoplasmosis rarely causes clinical disease in chickens^8^. Free-range chickens are one of the best indicators for soil contamination with *T. gondii* oocysts because they feed from the ground. Tissues of *T. gondii*-infected free-range chickens are considered good sources of infection for humans and other animals^8^. Together with the high prevalence of *T. gondii* infection in adult sheep and lambs and the various degrees of prevalence in pigs and cattle^3^,^8^, controlling *T. gondii* infection in these animals is crucial for controlling the disease transmission and vaccination is the most effective and economic method^9^.

The surface antigen 1 of *T. gondii* (TgSAG1), a major surface antigen of the infective tachyzoites, is considered the most promising candidate for a recombinant vaccine controlling *T. gondii* infection in mammals^3^,^9^. In mouse models, different types of recombinant vectors (e.g., *Neospora caninum, Salmonella typhimurium, Pseudorabies virus*, and *herpesvirus*) expressing TgSAG1 are said to possess efficient protective activities^10^,^11^,^12^,^13^. However, the protective efficacy of these TgSAG1-based recombinant vaccine against *T. gondii* infection in other mammals or birds is unclear.

*Eimeria tenella* is a closely related apicomplexan parasite of *T. gondii* that infects only chickens^14^,^15^. *E. tenella* is an emerging model to study the biological and immunological characteristics of the apicomplexan parasite^15^. With the establishment of transient and stable transfection systems in *Eimeria* parasites, *E. tenella* is being considered as a vaccine delivery vehicle carrying pathogen antigens, such as *Campylobacter jejuni* antigen A, that stimulate the protective immunity against *C. jejuni* infection in chickens^16^,^17^,^18^,^19^.

In the present study, we assessed the utility of *E. tenella* as a vaccine delivery vehicle by generating a line of transgenic *E. tenella* (Et-TgSAG1) expressing TgSAG1 and tested the capacity of this transgenic parasite to induce protective immunity against *T. gondii* infections in chickens and mice. We found that Et-TgSAG1 elicited TgSAG1-specific humoral and cellular immune responses in chickens, that reduced the *T. gondii* infection. Moreover, we detected TgSAG1-specific Th 1-dominant immune responses after intraperitoneal immunization with Et-TgSAG1 sporozoites in mice. The transgenic parasite-immunized mice showed a prolonged survival time compared with wild-type *E. tenella-* and non-immunized mice after challenge infection. Our encouraging results indicate that a transgenic *E. tenella* could provide a new tool for the production of a live recombinant vector vaccine against toxoplasmosis or other pathogens in both mammals and birds.

## Results

### Generation of transgenic *E. tenella* expressing TgSAG1 (Et-TgSAG1)

**Figure 1:**
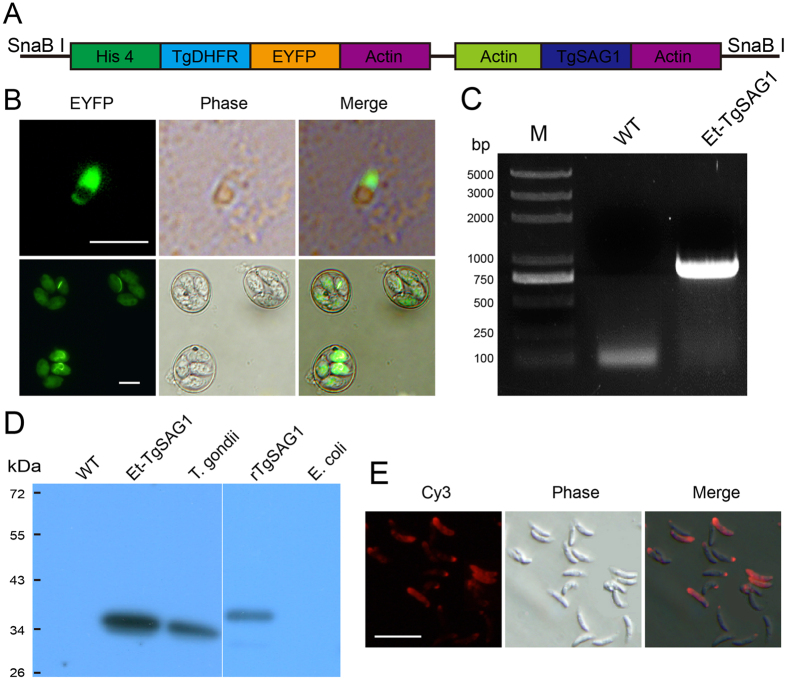
Generation of the transgenic *E. tenella* expressing TgSAG1. (**A**) The expression cassettes are shown as coloured boxes, and SnaB I was used to linearize the plasmid. (**B**) The fluorescent sporozoites (a) and oocysts (b) were detected after *in vitro* culture and *in vivo* passage, respectively. Bar = 10 μm. (**C**) Genomic DNA from Et-TgSAG1 was amplified with the primers SAG1-Age I-5 and SAG1-Sac II-3 (yielding a 1020-bp product) to verify the recombination of TgSAG1. Genomic DNA from wild-type *E. tenella* was used as a control. (**D**) TgSAG1 expression in the recombinant *E. tenella* was confirmed via western blot analysis with a TgSAG1-specific antibody, which detected a product at approximately 37 kDa. Wild-type *E. tenella* sporozoites antigen (WT), the *T. gondii* tachyzoite antigen, recombinant TgSAG1 purified from *E. coli* (rTgSAG1) and untransformed *E. coli* served as controls. (**E**) TgSAG1 expression and distribution in the Et-TgSAG1 sporozoites was confirmed via IFA with a TgSAG1-specific monoclonal antibody. Bar = 10 μm.

We transfected *E. tenella* sporozoites with the double expression-cassette plasmid pHDEAASAG1A, (Fig. 1A), which contained an N-terminal secretory signal sequence and the C-terminal GPI anchoring signal of TgSAG1 as surface-expressed antigens for easily inducing host immune responses^20^. We observed that approximately 0.2% sporozoites expressed enhanced yellow fluorescent protein (EYFP) 24 h after transfection *in vitro* (Fig. 1B). We obtained the stably transfected *E. tenella* oocysts, among which more than 90% of the excreted oocysts expressed EYFP (Fig. 1B), under the action of pyrimethamine and via fluorescence-activated cell sorting (FACS) following *in vivo* passage (Supplement Table 1).

We conducted several assays to ensure TgSAG1 expression in Et-TgSAG1. First, we confirmed that the TgSAG1 gene existed in the Et-TgSAG1 genome via PCR, as a TgSAG1-specific band was obtained from Et-TgSAG1 genomic DNA after PCR amplification (Fig. 1C). We also confirmed TgSAG1 expression in Et-TgSAG1 via western blot analysis. A specific band of approximately 37 kDa, which was similar in size to that of the native TgSAG1 expressed in *T. gondii*, was detected in Et-TgSAG1 (Fig. 1D). We further confirmed the distribution of TgSAG1 in the Et-TgSAG1 sporozoites by immunofluorescence assay (IFA), as we utilized its naïve regulators, both the N-terminal secretory signal sequence and the C-terminal GPI anchoring signal sequence. We found that TgSAG1 was expressed on the cell surface of Et-TgSAG1 sporozoites as its native distribution in tachyzoites of *T. gondii* (Fig. 1E). Taken together, we obtained a stably transfected *E. tenella* line with cell surface expression of TgSAG1 for investigating its immunogenicity and its potential application as a vaccine against toxoplasmosis.

### Et-TgSAG1 elicits TgSAG1-specific humoral and cellular immune responses in chickens

**Figure 2:**
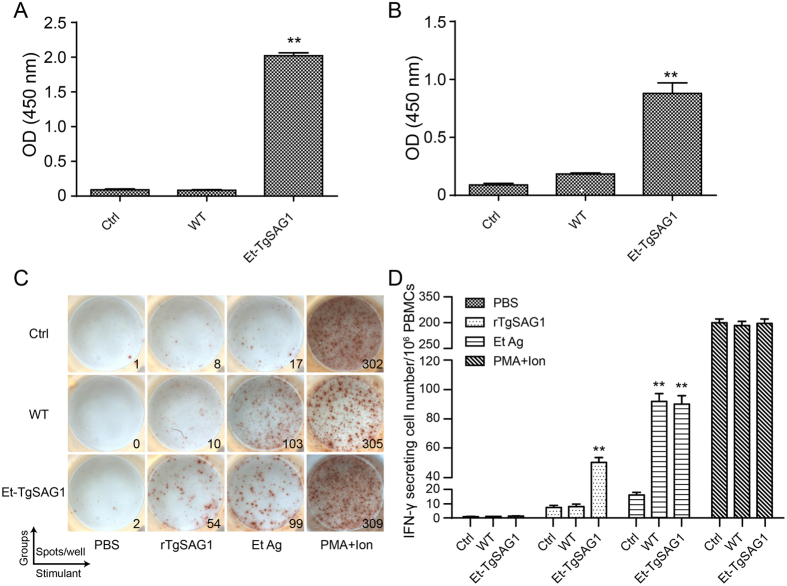
TgSAG1-specific humoral and cellular immune responses stimulated via Et-TgSAG1 oral vaccination in chickens. (**A**) The IgY antibody response to recombinant TgSAG1. TgSAG1-specific IgY antibody was detected via ELISA with a recombinant TgSAG1 expressed in *E. coli*. (**B**) The IgY antibody response to native TgSAG1. Native TgSAG1-specific IgY antibody was analysed via ELISA with the *T. gindii* tachyzoite antigens. Each bar represents the antibody levels of the sera from 6 chickens. (**C**) A total of 10^6^ PBMCs from naïve (Ctrl), wild-type *E. tenella* (WT)- and Et-TgSAG1 (Et-TgSAG1)-immunized birds were stimulated for 24 h with PBS, recombinant TgSAG1 (rTgSAG1), *E. tenella* oocysts antigen (Et Ag) and PMA plus ionomycin (PMA+Ion, positive control). The number of IFN-γ-secreting lymphocytes (spots) was detected as described in the Methods. (**D**) The mean amount of TgSAG1-specific IFN-γ secreting lymphocytes in the PBMCs in the Et-TgSAG1-immunized birds was significantly higher (p < 0.05) than that in the birds left naïve or immunized with the wild-type *E. tenella* (n = 6).

We analysed the TgSAG1-specific immune response after immunization to evaluate the immunogenicity of TgSAG1 expressed by Et-TgSAG1. We found that serum from Et-TgSAG1-immunized birds showed a significantly higher antibody titer against recombinant TgSAG1 than those of wild-type *E. tenella-*immunized and naïve birds (Fig. 2A). We also found that antibodies stimulated by Et-TgSAG1 recognized native TgSAG1, as a comparable antibody response against *T. gondii* tachyzoite antigens was detected (Fig. 2B).

Cell-mediated immunity (CMI) is the critical factor in the host defence against toxoplasmosis^3^,^11^. We analysed TgSAG1-specific CMI after vaccination, as revealed by the interferon (IFN)-γ secreting lymphocyte ratio in peripheral blood mononuclear cells (PBMCs) by Enzyme-Linked Immuiospot Assay (ELISPOT). We observed an increased number of TgSAG1-specific IFN-γ secreting lymphocytes in the PBMCs of the Et-TgSAG1 immunized birds compared to those of wild-type parasite-immunized and naïve birds (Fig. 2C,D). Taken together, these data demonstrate that, TgSAG1, expressed by the transgenic parasite with high immunogenicity, induced TgSAG1-specific humoral and cellular immune responses in chickens.

### Et-TgSAG1 elicits a Th 1-dominant immune response in mice

**Figure 3:**
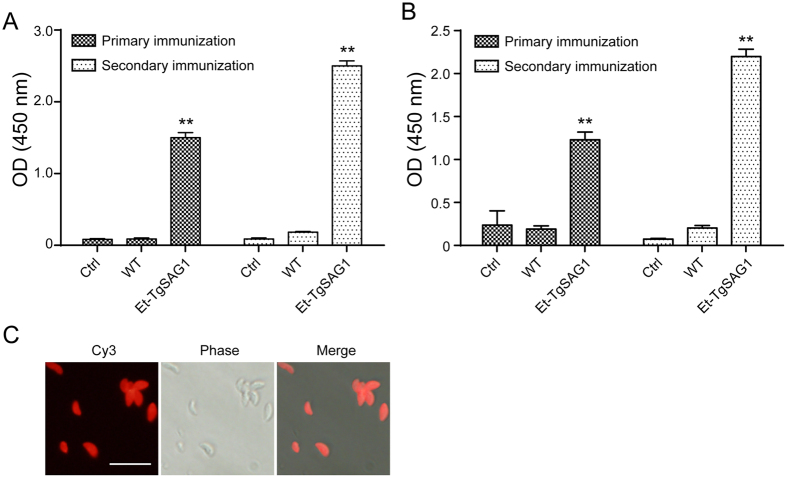
TgSAG1-specific humoral immune response stimulated by Et-TgSAG1 sporozoites in intraperitoneally immunized mice. (**A**) The IgG antibody response to recombinant TgSAG1 after the primary and boost immunizations. TgSAG1-specific IgG antibody was detected via ELISA with a recombinant TgSAG1 expressed in *E. coli*. (**B**) The IgG antibody response to native TgSAG1 after the primary and boost immunization. Native TgSAG1-specific IgG antibody was analysed via ELISA with *T. gondii* tachyzoite antigens. Each bar represents the antibody levels of the sera from 10 mice. (**C**) Antisera from Et-TgSAG1-immunized mice reacted with native TgSAG1. Indirect immunofluorescence staining of *T. gondii* tachyzoites with the antisera from the Et-TgSAG1-immunized mice. Bar = 10 μm.

*E. tenella* is an exquisitely host-specific pathogen that infects only chickens^14^. To test whether Et-TgSAG1 elicits an antigen-specific immune response in mammals, we intraperitoneally immunized BALB/c mice with Et-TgSAG1 sporozoites. We detected a TgSAG1-specific immune response after the primary immunization with Et-TgSAG1, and the immune response was boosted after a secondary immunization (Fig. 3A). In contrast, mice immunized with wild-type sporozoites did not generate antibodies against TgSAG1(Fig. 3A).

We coated the plates with *T. gondii* tachyzoite antigens to react with the sera from Et-TgSAG1-immunized mice to evaluate whether the generated antibodies could recognize the native TgSAG1. We found that the IgG antibody titer against the *T. gondii* tachyzoite antigens was distinctly higher in Et-TgSAG1-immunized mice than in the wild-type parasite-immunized or naïve mice (Fig. 3B). Additionally, the reaction was enhanced after the secondary immunization (Fig. 3B). Further evidence that the Et-TgSAG1-immunized mice generated antibodies recognized native TgSAG1 was that the *T. gondii* tachyzoites reacted with the sera from the Et-TgSAG1-immunized mice (Fig. 3C).

**Figure 4:**
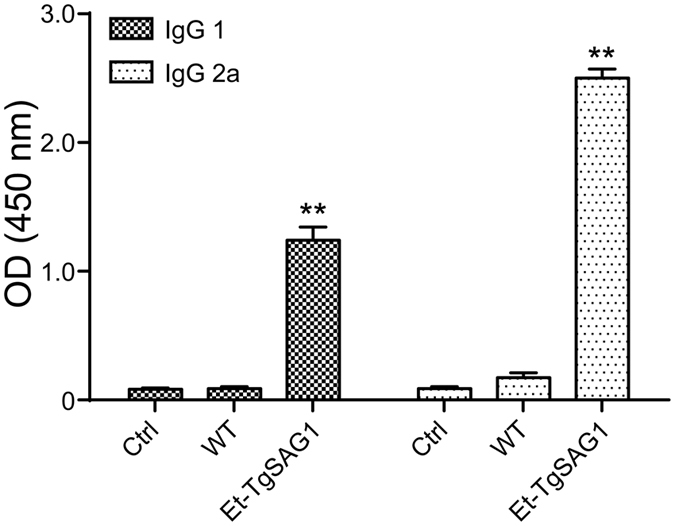
Th 1- and/or Th 2- type immune responses induced by Et-TgSAG1 sporozoites in intraperitoneally immunized mice. The levels of IgG 1 and IgG 2a antibodies to TgSAG1 2 weeks after the boost immunization were analysed via ELISA with recombinant TgSAG1 (n = 10).

The Th 1 immune response plays a major role in the host protective immunity against *T. gondii* infection in mice^3^,^9^. We analysed the distribution of the IgG subtypes, IgG 1 and IgG 2a, against recombinant TgSAG1 in serum after the boost immunization^9^,^11^. A mixed IgG 1/IgG 2a response with predominant IgG 2a production was detected in the sera of the mice immunized with Et-TgSAG1, indicating that Et-TgSAG1 elicited a Th 1-dominant immune response in mice (Fig. 4). These results demonstrated that TgSAG1, expressed by a chicken-specific pathogen, was recognized by the mouse immune system and elicited specific immune responses.

### Vaccination with Et-TgSAG1 partially protects chickens and mice against *T. gondii* infections

To evaluate the protective effects of Et-TgSAG1 applied as an anti-toxoplasmosis vaccine, we conducted a challenge infection with virulent *T. gondii* tachyzoites in Et-TgSAG1-immunized birds and mice. All the chickens were subsequently infected via intramuscular injection with *T. gondii* tachyzoites of the RH strain. We euthanized three chickens from each group at 1 -week post challenge infection and analysed the sizes of their spleens to determine the degree of inflammation, as there are rarely clinical symptoms of *T. gondii* infection in chickens. We found that the spleens of the naïve and wild-type *E. tenella-*immunized were enlarged after *T. gondii* infection compared with the Et-TgSAG1-immunized and non-challenged birds (Supplemental Fig. 1). These results demonstrated that Et-TgSAG1 immunization reduced the inflammatory reaction after *T. gondii* infection.

**Figure 5:**
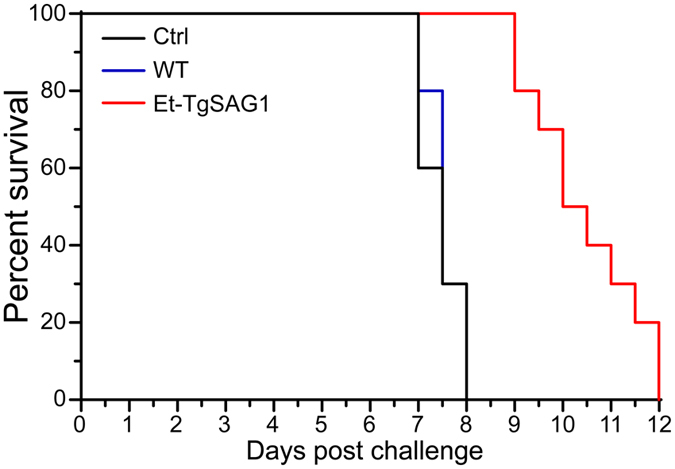
Protection of BALB/c mice against *T. gondii* infection. The survival curves of immunized BALB/c mice after lethal challenge with 50 tachyzoites of the virulent *T. gondii* RH strain 2 weeks after the secondary immunization.

We further analysed the protective effects of Et-TgSAG1 in mice. All the mice were challenge-infected with a lethal dose of *T. gondii* tachyzoites, and mouse survival was measured every 12 hours after the challenge infection. We found that the Et-TgSAG1-immunized mice showed a prolonged survival time compared with the wild-type *E. tenella*- and non-immunized mice after the challenge infection (Fig. 5). These data revealed that Et-TgSAG1 immunization provided partial protection against *T. gondii* infection in mice.

## Discussion

In this study, we constructed a transgenic *E. tenella* line (Et-TgSAG1) expressing an immunodominant antigen (TgSAG1) of *T. gondii* tachyzoites and demonstrated that Et-TgSAG1 elicits TgSAG1-specific humoral and cellular immune responses in chickens and Th 1-dominant immune responses in mice. More importantly, Et-TgSAG1 immunization provided partial protection against *T. gondii* infection in chickens and mice. These findings are encouraging and suggest the application of Et-TgSAG1 as a toxoplasmosis vaccine candidate both in birds and mammals.

The control of *T. gondii* infection in chickens is critical to block *T. gondii* infection transmission from chickens to other livestock or humans^8^. Coccidiosis caused by *Eimeria* spp. occurs in almost all poultry farms and free-range chickens^14^. Vaccination with either virulent or live attenuated live parasite formulations can efficiently protect chickens against *Eimeria* spp. infection^21^,^22^. *E. tenella* is one of the formulations used in anti-coccidial vaccines. Therefore, vaccination with a TgSAG1-based transgenic *E. tenella* as the formulation of the anti-coccidial vaccine also result in the reduction of *T. gondii* infection in chickens.

The genome size of *Eimeria* spp. is estimated to be between 55 and 60 Mbp, encoding 8,000–9,000 genes (http://www.genedb.org/Homepage/Etenella), which demonstrates *Eimeria* is a vaccine vector with large capacity. The adaptive immune system of the host recognizes a large number of antigens^23^. In the present study, considerable immune responses were detected against one exogenous gene product, TgSAG1, a cell surface expressed antigen, indicating that *Eimeria*-original antigens do not block immune responses against exogenous antigens expressed by transgenic *Eimeria*. Other studies with intracellular parasites, such as *E. tenella, T. gondii, Leishmania major*, and *Trypanosoma cruzi* expressing model antigens targeted to subcellular compartments have shown that secretory antigens were presented to and primed CD8 T cells^24^,^25^,^26^,^27^. When developing a recombinant vaccine vector expressing other pathogens antigens based on transgenic *E. tenella*, both the secreting and cell surface expressing exogenous antigens are optional as revealed in this study.

Sporozoites from *E. tenella* invade many types of cells *in vitro*, including cells from mice^28^,^29^,^30^. We also observed that the sporozoites of transgenic *E. tenella* are released in the mouse gut lumen and invade the epithelium cells but do not develop further (our unpublished data). This process may partly contribute to the mechanism that *E. tenella* (a host-specific parasite) utilizes as a vaccine vector while carrying TgSAG1 to elicit heterogeneous antigen-specific immune responses in mice.

Et-TgSAG1 immunization prolonged mouse survival after the challenge infection but did not provide full protection. Efforts should be focused on exploring strategies for enhancing the protective immunity elicited by transgenic *E. tenella* to realize the utilization of the parasite as a powerful vaccine delivery vehicle. These strategies may include but are not limited to following: 1) improving the relative magnitude of the exogenous antigens in transgenic *Eimeria* spp^31^; 2) co-expression of the antigens with cytokines, such as interleukin 2 (IL-2), or immune receptor ligands, such as CD40L or flagellin, as adjuvants^32^,^33^,^34^ and 3) optimizing the immunization schedule^35^. In addition, recombinant vaccines providing full protection can hardly be realized based on only one immunodominant antigen against *T. gondii,* a complex parasitic infection. Efforts also should be focused on co-expressing multiple immunodominant antigens of *T. gondii* such as dense granule (GRA) molecules or rhoptry proteins in transgenic *E. tenella*^36^,^37^. This proposal can be easily realized using our established tactics with double or multiple expression cassettes or with a single expression cassette mediated by P2A^38^,^39^.

*Eimerian* parasites, such as *E. tenella,* are exquisitely host-specific and do not complete their life cycle in other animals^28^,^29^,^30^. Therefore, it is very safe to use *E. tenella* as a delivery system to induce an immune response in mammals. The results of this study further established the feasibility of using *E. tenella* as a delivery system for priming immune responses against heterologous pathogens in chickens and mammals.

## Methods

### Ethics statement

All animal experiments were performed in strict accordance with the China Agricultural University Institutional Animal Care and Use Committee guidelines and followed the International Guiding Principles for Biomedical Research Involving Animals. The experiments were approved by the Beijing Administration Committee of Laboratory Animals.

### Parasites and animals

*E. tenella* (BJ strain) was maintained and propagated in coccidia-free, 3-week-old AA broilers. The oocysts were collected from the faeces of chickens 6–9 days post infection (dpi) and were isolated, purified, and sporulated as described previously^40^. Sporozoite excystation and purification were achieved using a previously described method^41^.

The RH strain of *T. gondii* was maintained by serial passages in African green monkey kidney (VERO) cells in DMEM supplemented with foetal bovine serum (FBS; 10% v/v), penicillin (200 U ml^−1^) and streptomycin (20 mg ml^−1^) in a humidified atmosphere of 5% CO_2_ at 37 °C^42^.

Female BALB/c mice were purchased from the Academy of Military Medical Sciences Laboratory Animal Center (Beijing, China). All mice were maintained under specific-pathogen-free conditions and were at 6–8 weeks of age when the immunizations were initiated. The mice were acclimated for approximately 7 days prior to the start of the experiment and were weighed (weight >20 g) to establish a baseline for detecting any reductions in body weight caused by the infection.

Three-week-old SPF chickens were purchased from Merial Animal Health Co., Ltd. (Beijing, China) They were housed in coccidian-free isolators and were fed a pathogen-free diet and water *ad libitum*.

### Cloning and expression of the TgSAG1 gene and transfection plasmid construction

Total RNA was isolated from 1 million *T. gondii* tachyzoites using TRIzol reagent (Invitrogen, USA) cDNA was synthesized using random primers and a High Capacity cDNA Reverse Transcription Kit (Applied Biosystems)^24^. The TgSAG1 open reading frame (ORF) (GenBank: JX045421.1) was PCR-amplified from *T. gondii* tachyzoite cDNA using primers with introduced Age I and Sac II sites (underlined): SAG1-Age I-5: 5′-ACCGGTATGTCGGTTTCGCTGCACCAC-3′; SAG1-Sac II-3: 5′-CCGCGGTCACGCGACACAAGCTGCG-3′.The amplified fragment was inserted into the pEASY-Blunt Simple Cloning Vector (TransGen Biotech, Beijing, China) and sequenced. The TgSAG1 ORF without the signal sequence and the C-terminal GPI anchoring sequence was amplified using the primers SAG1-EcoR I-5 (5′-GAATTCATGGCAGGGGTGTTTGCCGCGCCC-3′) and SAG1-Xho I-3 (5′-CTCGAGCCCTGCAGCCCCGGCAAACTCC-3′), and the resulting fragment, bearing the EcoR I and Xho I restriction sites (underlined), was cloned into the multiple cloning site of pET-28a between the EcoR I and Xho I restriction enzyme sites. All PCR amplifications were performed using the high-fidelity thermostable *Pfu* DNA polymerase to reduce the mutation frequency. The vector was transformed into *E. coli* for protein expression. The recombinant 6x His tagged proteins were purified from the soluble fraction of the lysate using a Hi-Trap metal-chelating column (GE Healthcare, USA) The identity and purity of the proteins were evaluated via SDS-PAGE in 12% polyacrylamide gels and western blot analysis.

The double expression-cassette plasmid, pHDEAASAG1A, was constructed based on the pHDEAAssChIL-2A plasmid^34^,^38^. Briefly, the ChIL-2 gene was replaced by the Age I- and Sac II- digested TgSAG1 ORF from the pEASY-Blunt Simple Cloning Vector carrying the entire TgSAG1 ORF. The resulting plasmid, was sequenced to confirm its integrity as described above. Plasmid DNA was purified using the Qiagen Endo Free Plasmid Maxi Kit (Qiagen, Germany) as described by the manufacturer.

### Transfection and selection of transgenic parasites

The linearized plasmids, digested with the SnaB I restriction enzyme, were transfected into the *E. tenella* sporozoites by restriction enzyme-mediated integration (REMI) as previously described^34^,^43^. We inoculated half of the electroporated sporozoites (5 million) into primary chicken kidney cells (PCKCs) for observing the transient transfection ratio *in vitro*^34^,^43^. The other half of the transfected sporozoites was inoculated equally into the ileocecal openings of five 3-day-old chickens via the cloaca for stable transfection selection. Eighteen hours after the inoculation, a standard diet supplemented with 150 ppm pyrimethamine (Sigma, USA) was given. Oocysts in the faeces, between days 6 and 9 post-inoculation, were collected and checked using a fluorescence microscope^18^,^38^. Transfected oocysts expressing EYFP were sorted in a MoFlo Cell Sorter (Dako Cytomation, Fort Collins, CO) in the single-cell mode after filtration through a 40-μm nylon cell strainer (BD Falcon, Boston, MA)^18^. The fluorescent oocysts were continuously propagated *in vivo* under the action of pyrimethamine (150 ppm) until more than 90% of the oocysts were expressing in the population (Supplemental Table 1).

### Indirect immunofluorescence assay (IFA)

Freshly extracted Et-TgSAG1 sporozoites and tachyzoites of the *T. gondii* RH strain were washed in phosphate-buffered saline (PBS), air-dried on poly-L-lysine slides, and fixed in acetone^24^,^34^. The slides were incubated at 37 °C with an anti-TgSAG1 monoclonal antibody (1:1000, a gift from Dr Dominique Soldati) or sera (1:100) from the Et-TgSAG1-immunized mice for 1 h. The antibodies bound to the *E. tenella* parasites and *T. gondii* tachyzoites were detected with Cy3-conjugated goat anti-mouse IgG (1:200, Proteintech Group Inc., Chicago). The slides were washed in PBS and analysed using a fluorescence microscope (Olympus IX71, Tokyo, Japan).

### Western blot analysis

For the western blot assay, the total lysates were prepared from the purified Et-TgSAG1 sporozoites and were subjected to SDS-PAGE in 12% polyacrylamide gels. The separated proteins were transferred to a polyvinylidene difluoride (PVDF) membrane (Millipore, USA) and were probed with an anti-TgSAG1 monoclonal antibody (1:2000). A secondary peroxidase-conjugated goat anti-mouse IgG antibody (1:200, Proteintech Group Inc., Chicago) was used for detction. Proteins were visualized with ECL chemiluminescence reagents (CoWin Biotech Co., LTD., Beijing, China)^34^,^44^. Recombinant TgSAG1(rTgSAG1) and soluble proteins extracted from *T. gondii* tachyzoites served as positive controls, while the wild-type *E. tenella* sporozoites (WT) and *E. coli* soluble antigens served as negative controls.

### Vaccination and challenge infection in chickens

Groups of six inbred SPF chickens were either left naïve (Ctrl) or were immunized via oral infection with 5000 sporulated wild-type *E. tenella* (WT) and Et-TgSAG1 (Et-TgSAG1) oocysts at the age of 3 weeks. The inoculation solution of each chicken was 300 μl of sterile PBS from the naïve mice or 300 μl of sterile PBS containing 5000 sporulated oocysts from the wild-type- or transgenic *E. tenella-*immunized chickens. The chickens were housed in coccidian-free isolators and were fed a pathogen-free diet and water. Serum samples and PBMCs were collected from the chickens at 2 and 4 weeks after immunization for analysing the humoral and cellular immune responses via ELISA and ELISPOT, respectively.

The ELISA was performed as previously described^34^. In brief, microtiter plates were coated with recombinant TgSAG1 (200 ng/well) and *T. gondii* tachyzoites antigens (500 ng/well) in 50 mM carbonate buffer (pH 9.6). The chicken sera were diluted to 1:100 with PBST containing 2% skim milk and were applied to the wells. The horseradish peroxidase (HRP)-conjugated goat anti-chicken IgY Fc fragment (Bethyl Laboratories, Inc.) was diluted to 1:5000 with PBST containing 2% skim milk and was used as the secondary antibody. The ELISA was developed using 3,3′,5,5′-tetramethylbenzidine (TMB) and H_2_O_2_ as the substrates, and the optical density was read at 450 nm (OD 450) with an ELISA reader (Bio-TekEL 680, USA).

The ELISPOT was conducted to assay the TgSAG1-specific cellular immune response following the previously described protocols^34^,^45^. Briefly, 10^6^ PBMCs from the naive, wild-type *E. tenella* (WT) and Et-TgSAG1 oocyst-immunized birds were stimulated overnight at 37 °C in 5% CO_2_ with 10 μl PBS, 2 μg recombinant TgSAG1 (rTgSAG1), 10 μg *E. tenella* oocyst antigen (Et Ag) and 10 μl phorbol 12-myristate 13-acetate (PMA) plus ionomycin (PMA + Ion, 10 ng/ml PMA plus ionomycin 5 μg/ml). The spots, which represented IFN-γ-secreting lymphocytes, were detected 24 h after the stimulation, as previously described^34^,^45^. The cells in the plate were completely removed and washed. The wells were subsequently incubated with 1 μg/ml biotinylated detection antibody (Biosource International, USA) and a streptavidin-HRP-conjugated secondary antibody (Biosource International, USA) Then, each well of the plate was treated with 100 μl of 3-amino-9-ethylcarbazole (AEC) substrate solution (Dakewei, China) and was incubated at room temperature for 30 min in the dark. The spots in each well were counted using an automated ELISPOT reader (Bioreader 4000; Bio-sys, Germany).

To test whether Et-TgSAG1, used as an anti-toxoplasmosis vaccine, protects chickens against *T. gondii* infection, all chickens immunized with or without Et-TgSAG1 or its wild-type parasite were subsequently challenged via intramuscular injection with 1 × 10^6^ *T. gondii* tachyzoites of the RH strain at 4 weeks post-immunization. Three chickens from each group were euthanized at 1-week post-challenge infection. Their spleen sizes were analysed to reveal the degree of inflammation after the *T. gondii* infection.

### Vaccination and challenge infection in mice

Groups of ten BALB/c mice were either left naïve (Ctrl) or vaccinated via two intraperitoneal injections at 2-week’ intervals with 1 × 10^6^ wild-type *E. tenella* sporozoites (WT) or Et-TgSAG1 sporozoites (Et-TgSAG1). The injection solution of each mouse was 200 μl sterile PBS from the naïve mice or 200 μl sterile PBS containing 1 × 10^6^ sporozoites from the wild-type or transgenic *E. tenella*-immunized mice. All mice were maintained under specific-pathogen-free conditions and were fed a pathogen-free diet and water. The mouse sera were collected from the tail vein at 2 weeks after the primary and secondary immunizations and were analysed by ELISA. The ELISA protocols were conducted as well as in the above description, except the secondary antibodies used in these assays were an HRP-conjugated goat anti-mouse IgG (Proteintech Group Inc., Chicago) and an HRP-conjugated goat anti-mouse IgG 1 or IgG 2a (Proteintech Group Inc., Chicago) for the isotype analyses. The secondary antibodies in this assay were diluted to 1:3000.

To test whether Et-TgSAG1 protects mice from a virulent *T. gondii* infection, all mice were subsequently challenged via intraperitoneal injection with 50 *T. gondii* tachyzoites of the RH strain 2 weeks after the secondary immunization. Mouse survival was measured every 12 hours after the challenge infection. We paid special attention to the welfare of the animals after challenge infection, and the mice were euthanized when the mice lost more than 20% of their body weight due to illness caused by *T. gondii* infection at each time point of the observation.

### Statistical analyses

The experiments were analysed using GraphPad Prism 5.0 d (GraphPad Software), and the differences between the control and the treated groups were analysed using SPSS 12.0 (SPSS Institute Inc.). The differences in the experimental treatments were tested using Duncan’s Multiple Range Test following ANOVA with significance reported at P ≤ 0.05.

## Additional Information

**How to cite this article**: Tang, X. *et al*. Transgenic *Eimeria tenella* as a vaccine vehicle: expressing TgSAG1 elicits protective immunity against *Toxoplasma gondii* infections in chickens and mice. *Sci. Rep.* **6**, 29379; doi: 10.1038/srep29379 (2016).

## Supplementary Material

Supplementary Information
