# Supplementary material for: Transgenic Eimeria tenella as a vaccine vehicle: expressing TgSAG1 elicits protective immunity against Toxoplasma gondii infections in chickens and mice
Source: Sci Rep. 2016 Jul 8;6:29379. doi: 10.1038/srep29379 (PMC4937369; doi:10.1038/srep29379)
Supplement: Supplementary Information [file srep29379-s1.pdf]

Transgenic *Eimeria tenella* as a vaccine vehicle: expressing TgSAG1 elicits protective immunity against *Toxoplasma gondii* infections in chickens and mice

Xinming Tang[1], Guangwen Yin[2], Mei Qin[1], Geru Tao[1], Jingxia Suo[1], Xianyong Liu[1], Xun Suo[1,*]

[1] State Key Laboratory of Agrobiotechnology & Key Laboratory of Zoonosis of Ministry of Agriculture & National Animal Protozoa Laboratory, College of Veterinary Medicine, China Agricultural University, Beijing, 100193, China

[2] Engineering Laboratory of Animal Pharmaceuticals, College of Animal Science, Fujian Agriculture and Forestry University, Fuzhou, 350002, Fujian Province, China

* Corresponding author: Xun Suo Tel: +86-10-62734325.

E-mail address: suoxun@cau.edu.cn

Supplement Table 1. Stably transfected *Eimeria* parasite (Et-TgSAG1) selection.

Reporter	Generation	Inoculate dosage (Oocysts/bird)	% Parasite expressing	Oocysts output/bird	Next selection
EYFP	1	$1×10^6$ (sporozoites)	1.1	$1.5×10^3$	Drug+FACS
	2	100	13.8	$7.8×10^6$	Drug+FACS
	3	5000	42.3	$2.0×10^7$	Drug+FACS
	4	5000	78.2	$3.5×10^7$	Dug+FACS
	5	5000	92.1	$3.2×10^7$	---
	6	5000	91.3	$5.6×10^7$	---
	7	5000	90.6	$4.9×10^7$	---

Supplement Figure 1. The size of chicken spleens after challenged with *T. gondii* tachyzoites.

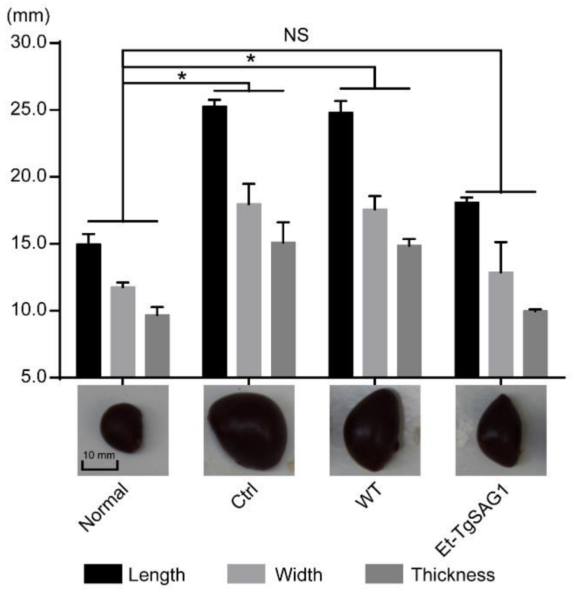

Supplement Figure 1. The size of chicken spleens after challenged with *T. gondii* tachyzoites. The length, width and thickness of the spleens were measured at 1-week post challenge infection. The spleen size of naïve chickens without challenge infection (Normal) served as control. NS: No significant difference. Bar=10 mm.
